# A Manual of Pathological Anatomy

**Published:** 1855-11

**Authors:** 


ART. VII.—*A Manual of Pathological Anatomy.* By CARL ROKITANSKY, M. D., Curator of the Imperial Pathological Museum, and Professor at the University of Vienna, etc. Translated from the last German Edition by WILLIAM EDWARD SWAIN, M. D., EDWARD SIEVEKING, M. D., CHAS. HEWITT MOORE, GEORGE E. DAY, M. D., F. R. S. Four volumes in two. Philadelphia: Blanchard & Lea. 1855.

The great work of the great pathologist has hitherto been a sealed book to the English reader. Its translation and republication had been a task several times attempted, and as often resulting in failure. Finally that noble institution, the Sydenham Society, took it up, and assigned a volume to each of the four gentlemen whose names appear as translators on the title-page. First volume 2 appeared, then volumes 3 and 4, and, ending with the beginning, in this year the first volume was completed.

Messrs. Blanchard & Lea, with a commendable promptness, have reissued it for American readers, condensing, for the sake of cheapness, the four volumes into two, a change, however, which involves only the binding and not the matter of the work.

The profession is too well acquainted with the reputation of Rokitansky to need our assurance that this is one of the most profound, thorough, and valuable books ever issued from the medical press. It is *sui generis*, and has no standard of comparison. We shrink from the task of attempting a review of these twelve hundred closely printed pages, for were we to essay a careful notice few would thank us for our pains. It is only necessary to announce that it is issued in a form as cheap as is compatible with its size and preservation, and its sale follows as a matter of course. No library can be called complete without it.

One word of the author himself and we leave the book to the pockets of our readers: "Charles Rokitansky, the founder of the German (it should rather have been called Austrian) medico-anatomical school, was born at Königsgrastz, in Bohemia, was educated at the Gymnasium of Leitneritz, and graduated at Vienna, in 1828. Shortly afterward he was appointed assistant in the patholagico-anatomical department of the University, and in 1854 Professor of Pathological Anatomy. At the same time he was instituted Prosector at the General Hospital, at Vienna, and also sole medico-legal Anatomist for the examination of all doubtful cases of death throughout that metropolis.

"The immense fund of materials thus placed at his disposal (the number of corpses dissected is summed up at 30,000) was almost entirely reserved for the elaboration of that grand work on pathological anatomy, which, in the consciousness of having thoroughly mastered the subject, he gave to the world between the years 1842 and 1846; which has passed, *unaltered*, through three reimpressions; and which, under the auspices of the Sydenham Society, has been translated into the English language.

"In 1849, Rokitansky was appointed Dean of the Medical Faculty, and, in 1850, Rector of the University of Vienna."

